# Selenium enhances photodynamic therapy of C-phycocyanin against lung cancer via dual regulation of cytotoxicity and antioxidant activity: Selenium enhances PDT effect of PC anti-tumor activity

**DOI:** 10.3724/abbs.2023159

**Published:** 2023-11-22

**Authors:** Jie Shen, Haidong Xia, Xiaojing Zhou, Lei Zhang, Qian Gao, Kan He, Dahai Liu, Bei Huang

**Affiliations:** 1 School of Life Sciences Anhui University Hefei 230601 China; 2 Center for Stem Cell and Translational Medicine Anhui University Hefei 230601 China; 3 School of Medicine Foshan University Foshan 528000 China

**Keywords:** selenium, phycocyanin, PDT, ROS, antioxidant

## Abstract

As a natural photosensitizer, phycocyanin (PC) has high efficiency and uses low-intensity irradiation. To enhance the photodynamic therapy (PDT) of PC, we extract selenium-enriched phycocyanin (Se-PC) from Se-enriched *Spirulina platensis* and examine the synergistic effect of PC combined with selenium against lung tumors. *In vitro* experiments reveal that Se-PC PDT more efficiently reduce the survival rate of mouse lung cancer cells (LLC cell line) than PC PDT treatment by increasing the level of ROS and decreasing the level of GPx4, which is confirmed by the Chou-Talalay assay. *In vivo* imaging system analysis reveal that tumor volume is more markedly decreased in both the Se-PC PDT and PC PDT plus Na _2_SeO _3_ groups than in the PC PDT group, with inhibition rates reaching 90.4%, 68.3% and 53.1%, respectively, after irradiation with 100 J/cm ^2^ laser light at 630 nm. In normal tissues, Se-PC promotes the synthesis of antioxidant enzymes and the immune response by the IL-6/TNF-α pathway against tumor proliferation and metastasis. Using Se-PC as a photosensitizer in tumors, apoptosis and pyroptosis are the primary types of cell death switched by Caspases-1/3/9, which is confirmed by TEM. Based on the transcriptome analysis, Se-PC PDT treatment inhibits angiogenesis, regulates inflammation by the HIF-1, NF-κB and TGF-β signaling pathways and dilutes tumor metabolism by reducing the synthesis of glucose transporters and transferrin. Compared to PC PDT, Se-PC increases the expression levels of some chemokines in the tumor niche, which recruits inflammatory cells to enhance the immune response. Our study may provide evidence for Se-PC as an effective photosensitizer to treat lung cancer.

## Introduction

Despite advances in the prevention, diagnosis and treatment of lung cancer, this malignancy remains the leading cause of cancer-related death worldwide [1]. There are many methods for treating lung cancer, among which photodynamic therapy (PDT) has many advantages compared to traditional surgical treatment, such as small wounds, repeatability, and strong specificity [2]. PDT employs nontoxic dyes called photosensitizers (PSs) [3]. In the presence of molecular oxygen, photochemical reactions produce a variety of reactive oxygen species (ROS), including singlet oxygen and hydroxyl radicals. These ROSs may cause oxidative damage to proteins, lipids and nucleic acids, leading to cell death by necrosis or apoptosis [4] .

Phycocyanin (PC) is a water-soluble oligomeric protein with a light-capturing pigment consisting of phycobilin that can efficiently transfer captured light energy to the photoreaction center through fluorescence resonance energy transfer [5]. Degradative PC peptides can supply nutrients through the blood circulation in normal tissues. As a natural photosensitizer, phycocyanin has high efficiency and uses low-intensity irradiation at approximately 620 nm [6]. More importantly, PC is a tumor-associated macrophage (TAM)-targeted photosensitizer that binds to scavenger receptor-A [7]. Nevertheless, compared to small photosensitizer molecules, the PC structure is insufficiently stable to maintain effective PDT activity [8]. Our previous work has shown that PC PDT with selenium could significantly suppress tumor growth. [9].

Selenium is a trace element that exerts antitumor effects in multiple directions, including enhancing the activity of antioxidant enzymes [10]. High concentrations of selenium induce cytotoxicity to tumors, regulate inflammation and immune responses [11], prevent angiogenesis [12], and activate or inactivate key regulatory proteins of cell proliferation [13]. When selenium is used in combination with other medicines or therapies [14], it not only improves the antitumor toxicity of drugs but also reduces the toxicity and side effects of drugs on the body and the drug resistance of tumors. However, there are no reports regarding the synergistic effect of phycocyanin photosensitizers combined with selenium against lung cancer.

The He-Ne laser (wavelength of 632.8 nm) is characterized by high intensity and has several biological effects, such as increasing cell viability, improving phagocytosis, and promoting immune responses [15].

In the present study, we explored the synergistic effect and mechanism of Se-PC photosensitizers in tumor-bearing mice irradiated with a He-Ne laser by analyzing the cell death type, gene expression and tumor immunity. *In vivo* assays further demonstrated that Se-PC PDT could sufficiently suppress tumor growth, exhibiting great therapeutic potential.

## Materials and Methods

### Materials

DMEM and fetal bovine serum (FBS) were purchased from Gibco (Carlsbad, USA). The mouse Lewis lung carcinoma with luciferase-labelled LLC and LLC-luc cells were purchased from Hunan Fenghui Biotechnology Co., Ltd (Changsha, China).

A He-Ne laser was purchased from Nanjing Latron Laser Company (Nanjing, China), the transmission electronic microscope (TEM) was a Jem100sx from JEOL (Tokyo, Japan), and a Tanon-5200 multifunction imager was purchased from Shanghai Tianneng Technology Co., Ltd (Shanghai, China).

The assay kits for superoxide dismutase (SOD), glutathione peroxidase (GSH-Px), and malondialdehyde (MDA) were purchased from Nanjing Jiancheng Bioengineering Institute (Nanjing, China). ELISA kits for tumor necrosis factor-α (TNF-α) and interleukin-6 (IL-6) were purchased from Shanghai Fanke Biotechnology Co., Ltd (Shanghai, China). Adult C57BL/6 mice were obtained from ZiYuan (Hangzhou, China).

### Purification of selenium-enriched phycocyanin

The cultivation of *Spirulina platensis* was carried out in Zarrouk medium (pH 9.0) at 20°C under light illumination (4000 Lux; 14 h /10 h light/dark cycle). The culture of Se-enriched *S*. *platensis* was performed using a stepwise Se addition method by which Na _2_SeO _3_ was added to the medium on day 7 (100 mg/L), day 8 (150 mg/L, and day 9 (200 mg/L) for a cumulative concentration of 450 mg/L (OD _560_=1.2–1.3).

The dry powder was added to phosphate buffer (PB), frozen overnight and then centrifuged at 10,000 *g* for 25 min. The collected supernatant was added step by step to two different concentrations of (NH _4_ ) _2_SO _4_ (20% and 50%), centrifuged at 10,000 *g* and dialyzed overnight. Sephadex G-200 chromatography columns with linear NaCl gradients were used to filter the suspension. Selenium-enriched phycocyanin (Se-PC) was collected with a purity ratio (OD _620_/OD _280_ )>3 [9]. The absorption spectrum was detected by a UV-1901 spectrophotometer (Persee, Beijing, China), and the fluorescence spectra were recorded on a fluorescence spectrophotometer (Hitachi, Tokyo, Japan). The electrophoretic purity of Se-PC was analyzed by SDS-PAGE and native PAGE.

The Se-PC and PC powders were dissolved in a mixture of strong acid (HClO _4_ :HNO _3_; v:v=4:1) for heating digestion. The main functional elements of Se-PC and PC were determined by inductively coupled plasma-mass spectrometry (ICP-MS).

### Cell PDT treatment and combination index assay

LLC cells (5.0×10 ^3^ cells/well) were seeded in 96-well plates and exposed to different concentrations of PC or Se-PC for 12 h prior to 9 min of 26 J/cm ^2^ laser irradiation. After irradiation, the MTT assay was used to determine the survival rate [16].

LLC cells were treated with different concentrations of PC and Na _2_SeO _3_ alone or in combination for 12 h. The PDT irradiation dose and MTT assay were performed as described above. The combination index was calculated using the software Compusyn1.0, including synergistic effect (combination index<1), additive effect (=1) and antagonism (combination index>1) [17].

### Reactive oxygen species (ROS) detection

Intracellular ROSs were measured using DCFH-DA as a fluorescent probe. LLC cells (5×10 ^3^ cells/well) were cultured in 96-well plates and treated with PC-PDT, PC+Na _2_SeO _3_-PDT and Se-PC PDT. After incubation, the cells were exposed to DCFH-DA (20 μM) for 30 min at 37°C away from light. The fluorescence intensity was measured at an excitation wavelength of 488 nm and an emission wavelength of 525 nm, harvested, counted and the fluorescence value was calculated for every 10,000 cells.

### PDT treatment of mice

Mice were randomly divided into five groups with 5 mice per group: control, laser only, Se-PC PDT treatment that was administered with 0.2 mL of Se-PC (15 mg/mL), PC PDT or PC PDT plus Na _2_SeO _3_ treatment that were treated at the same dose. The selenium content of Na _2_SeO _3_ was equal to that of the selenium-rich phycocyanin group. These agents were injected beneath the tumors every 3 days and irradiated at a dose of 100 J/cm ^2^ using a He-Ne laser (630 nm, 45 mW/cm ^2^ ). The duration of drug treatment was 11 days. The control and laser groups were administered with the same volume of normal saline.

### Construction of the mouse model with lung tumors

Adult C57BL/6 male mice (18‒20 g) were used to construct tumor-bearing mice using subcutaneous injections of 1 × 10 ^6^ LLC-luc cells near the armpit area.

### *In vivo* imaging observation

During treatment, the body weight and tumor volume of each mouse were recorded every day. Eleven days later, drug was withdrawn, and tumor-bearing mice were intraperitoneally injected with a 150 mg/kg solution of fluorescein potassium salt and then imaged by using a multifunction imager. After the mice were sacrificed, the tumor and organs were removed to examine the metastasis of fluorescein and calculate the tumor inhibition rate.

### Histological observation

The tumor was fixed in 4% (v/v) paraformaldehyde solution for 48 h, dehydrated in different grades of ethanol (30%, 70%, 90% and absolute) and embedded in paraffin wax. Sections (5 μm) of tumor were cut, dewaxed and dehydrated with different grades of ethanol, stained with hematoxylin and eosin (H&E), dehydrated, and mounted in neutral balsam. The sections were observed under a microscope (Leica, Wetzlar, Germany).

### Transmission electron microscopy (TEM)

Fresh tumor tissue was fixed in 2.5% glutaraldehyde solution for 2 h and then postfixed in osmium tetroxide. After being dehydrated, embedded and sectioned, sections were observed under a transmission electron microscope (Jem100sx).

### Activity detection of antioxidant enzymes and cytokines

Tissues were rinsed with saline solution, ground to a homogenate on ice and centrifuged at 2500 *g* for 10 min. The supernatant was tested using kits to determine the activities of malondialdehyde (MDA), superoxide dismutase (SOD), and glutathione superoxide dismutase (GSH-Px). The contents of IL-6 and TNF-α in serum were determined using ELISA kits.

### Western blot analysis

Cells and tissues were lysed and centrifuged at 4°C to remove the solid materials. The supernatant was separated bySDS-PAGE and transferred to polyvinylidene fluoride (PVDF) membranes (Millipore, Billerica, USA). Subsequently, membranes were blocked with TBS (pH 7.4) containing 5% skim milk powder, and then incubated with the primary antibody overnight at 4°C, followed by incubation with the appropriate secondary antibody at room temperature for 2 h. Finally, the results were detected using an ECL kit (Thermo Fisher, Waltham, USA) and analyzed using ImageJ software. Protein levels were standardized to GAPDH levels. The primary antibodies used are as follows: anti-GPx-1 (381571; 1:1000; Zenbio, Chengdu, China), anti-GPx-4 (381958; 1:1000; Zenbio), anti-Caspase-1 (342947; 1:800; Zenbio), anti-Caspase-3 (R22842; 1:1000; Zenbio), anti-Caspase-8 (222121; 1:500; Zenbio), anti-Caspase-9 (381336; 1:1000; Zenbio), anti-Bcl-2 (26593-1-AP; 1:3000; Proteintech, Chicago, USA), and anti-GAPDH (60004-1-Ig; 1:50000; Proteintech). HRP-conjugated secondary antibodies (SA00001-1; 1:5000) were purchased from Proteintech.

### RNA extraction and sequencing

According to the manufacturer’s instructions, total RNA from 0.1 g tumor tissue was extracted using Trizol (Invitrogen, Carlsbad, USA) from each group, including the laser group (Laser1 and Laser2), PC-laser group (PC-La1 and PC-La2), and Se-PC laser group (SePC-La1 and SePC-La2). The quality of the RNA was assessed by agarose electrophoresis, and the integrity of the extracted RNA was assessed with a NanoDrop spectrophotometer (Thermo Scientific). cDNA synthesis and RNA sequencing were performed by Shanghai Personal Gene Technology Co., Ltd (Shanghai, China). The samples were sequenced with Illumina HiSeq X10 (Illumina, San Diego, USA) using a specification of 6G per sample. Our sequencing data have been uploaded into the Gene Expression Omnibus (GEO) database ( https://www.ncbi.nlm.nih.gov/geo/), which is available under accession number GSE192635. Differentially expressed genes (DEGs) of the samples were analyzed using DESeq software, and DEGs were screened strictly based on the cut-off values of |log2 Fold change|>1 and *P* value<0.05. The Database for Annotation, Visualization and Integrated Discovery (DAVID) v6.8 was used to analyze the GO functional enrichment and KEGG signaling pathways of the DEGs [18].

### Expression detection of target genes determined by qRT-PCR

The primers for the target genes were designed using Primer5 and tested using NCBI BLAST, which are listed in Table 1, produced by Generalbio (Chuzhou, China). RT-PCR was performed using 1 μg total RNA as template according to the instructions of the kit to synthesize the first chain of cDNA for direct use in fluorescence quantitative PCR. The SYBR qPCR SuperMix Plus kit (Vazyme, Nanjing, China) was used to configure the reaction system needed for qRT-PCR and placed in the PikoReal Real-Time PCR instrument (Thermo Scientific) for quantitative detection.

**Table:** **Table 1** Sequences of primers used for qRT-PCR analysis

Name	Forward primer (5′→3′)	Reverse primer (5′→3′)
*Slc2a1*	ATCGTCGTTGGCATCCTTATT	TGCAGGTCTCGGGTCACATC
*Nfkbia*	GCCTCTATCCACGGCTACCT	TCAGCCCCACATTTCAACAA
*Ccl24*	GTGACCATCCCCTCATCTTGC	TTGTATGTGCCTCTGAACCCA
*Mmp13*	CTACCATCCTGCGACTCTTGC	GCATTTCTCGGAGCCTGTCA
*Ppbp*	TGCCCACTTCATAACCTCCA	TGCCATCAGATTTTCCAGCT
*Vegfa*	GCTACTGCCGTCCGATTGA	CCTATGTGCTGGCTTTGGTGA
*Serpine1*	TAGCACAGGCACTGCAAAAGG	GATGACAAAGGCTGTGGAGGA
*Il1b*	TGCCACCTTTTGACAGTGATG	TGTGCTGCTGCGAGATTTG
*Ccl2*	CTGTGCTGACCCCAAGAAGG	GCTTGAGGTGGTTGTGGAAAA
*Ccl3*	CCCAGCCAGGTGTCATTTTC	GGCATTCAGTTCCAGGTCAGT
*Cxcl2*	CCACCAACCACCAGGCTACA	GCTTCAGGGTCAAGGCAAACT
*Cxcl12*	CAGTCAGCCTGAGCTACCGA	TGTTGTTCTTCAGCCGTGCA
*S100a9*	AGATGGAGCGCAGCATAACC	TGTGCTTCCACCATTTGTCTG
*Lcn2*	AGGCAATGCGGTCCAGAAA	TGGTTGTAGTCCGTGGTGGC
*Trf*	AAAACGGTCAAATGGTGCG	GAAGGACGGTCTTCATGTGGT
*GAPDH*	TGCCCAGAACATCATCCCT	GGTCCTCAGTGTAGCCCAAGA

### Statistical analysis

Data were analyzed using SPSS 22.0 and plotted using GraphPad Prism 5.0 software. Single-factor analysis of variance was used, and multiple comparisons were made using the Duncan test. *P*<0.05 was considered statistically significant.

## Results

### Determination of the characteristics of Se-PC

To analyze the characteristics of Se-PC, the content of the main functional elements was measured by ICP-MS, and the purity was determined by SDS-PAGE, native PAGE and spectrum analysis with a spectrophotometer.

As shown in Table 2, phycocyanin is a good selenium-enriched molecule, in which the content of selenium was concentrated from 0.068 mg/g of Se-SP to 0.86 mg/g of Se-PC, but other elements, such as Cr, Mn, and Zn, were reduced compared with PC. Meanwhile, Cr was concentrated from 0.002 mg/g SP to 0.022 mg/g PC, and the content of Zn remained almost unchanged in SP or PC, suggesting that selenium has a competitive advantage with phycocyanin compared to Cr and Zn.

**Table:** **Table 2** Main functional elements content in Se-PC or PC from *S*. *platensis*

Element (mg/g)	SP	PC	Se-SP	Se-PC
Se	0.002	0.031	0.068	0.86
Cr	0.002	0.022	0.006	0.004
Mn	0.025	0.009	0.01	0.005
Zn	0.430	0.440	0.34	0.18

**Figure 1:**
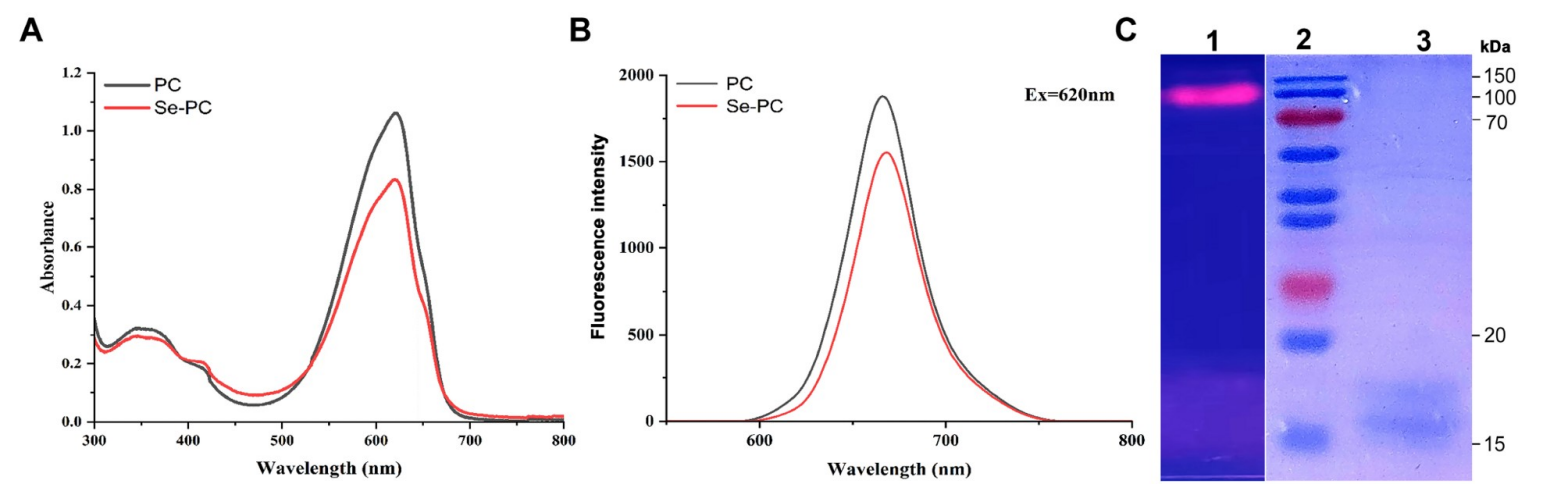
Figure 1 Spectral character and molecular weight analysis of Se-PC (A) Absorption spectra of Se-PC and PC. (B) Fluorescence spectra of Se-PC and PC. (C) SDS-PAGE and native-PAGE analysis of purified Se-PC. Lane 1: native PAGE, lane 2: protein markers, and lane 3: SDS-PAGE.

The Se-PC purified from Se-enriched Spirulina exhibited a single absorption peak at 620 nm, and the fluorescence peak was detected at 670 nm, which was similar to PC ( Figure 1A,B). As shown in Figure 1A, the spectral purity of Se-PC was 3.20 (OD _620_/OD _280_). SDS-PAGE analysis showed that the molecular weights of the Se-PC subunits were approximately 17.5 kDa and 18.5 kDa ( Figure 1C). Native PAGE showed that Se-PC had a molecular weight of approximately 108 kDa. This is similar to the electrophoretic purity of Se-PC reported in the literature.

### Se-PC PDT induced ROS generation in the LLC cell line

**Figure 2:**
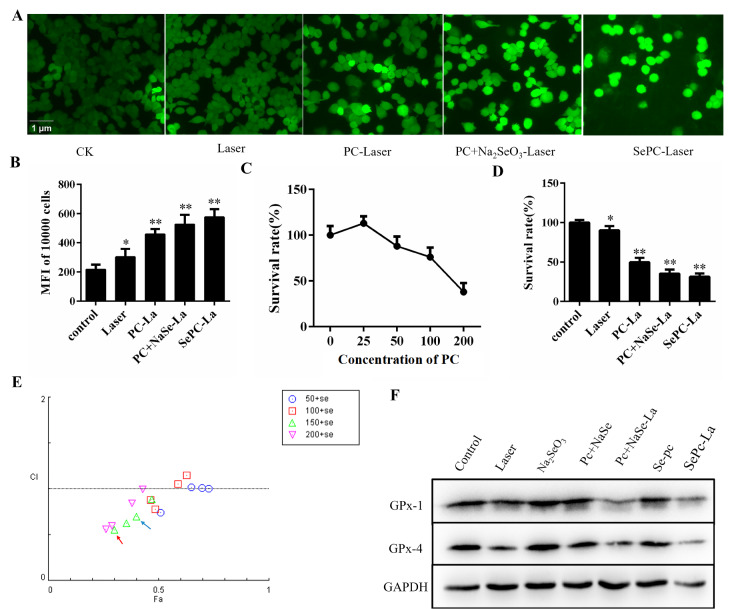
Figure 2 Se PC-PDT induced ROS generation in the LLC cell line (A) ROS levels in LLC cells, which were exposed to different concentrations of PC, PC+Na2SeO3 and Se-PC for 12 h after irradiation with a He-Ne laser, were detected by DCFH-DA-based assay. (B) The mean fluorescence intensity (MFI) was used to measure ROS levels. (C) Effect of Se-PC PDT on the survival rate of LLC cells at a concentration of 150 μg/mL. (D) Effect of PC PDT on the survival rate of LLC cells treated with different concentrations. (E) Drug combination index assay of PC and Na2SeO3 by the Chou-Talalay method. (F) GPX-1 and GPX-4 levels with different combination treatments between selenium and PC PDT.

To find a suitable concentration for PDT treatment, MTT method was used to assess the survival rate of PC PDT treatment with different concentrations. As shown in Figure 2C, the IC _50_ was 150 μg/mL with an irradiation dose of 26 J/cm ^2^; therefore, this concentration was used for the subsequent experiments. However, under this treatment condition, the survival rate of Se-PC PDT treatment reached 34% ( Figure 2D).

To investigate whether Se-PC PDT treatment could increase the ROS level in LLC cells, the DCFH-DA-based assay was used to detect the ROS level. As shown in Figure 2A, ROS accumulation was observed after irradiation for 12 h. We found that the ROS levels were increased to the highest levels with 150 μg/mL Se-PC PDT treatment compared to PC PDT and PC+Na _2_SeO _3_ PDT treatment ( Figure 2B), in which the content of selenium in Na _2_SeO _3_ was the same as the content in Se-PC (0.86 mg/g).

To confirm the synergistic effect of selenium (Na _2_SeO _3_) and PC PDT, the Chou-Talalay method was used to assess the combination index (CI) at different concentrations of PC (50‒200 μg/mL) and selenium (0.143‒1.14 μg/mL) with regard to its antitumor effect by the MTT method. As shown in Figure 2E, CI>1 indicated an antagonistic effect, CI=1 indicated an additive effect, and CI<1 indicated a synergistic effect. The best combination condition was 150 μg/mL PC+1.14 μg/mL Na _2_SeO _3_ (Se: 0.57 μg/mL) with a CI value of 0.56. In our study, the content of selenium in 150 μg/mL PC was 0.13 μg/mL with a CI value of 0.70, indicating that selenium could enhance the effect of PC PDT, and the PDT effect can also increase with increased concentrations of selenium up to 0.57 μg/mL.

GPX is a selenium-dependent enzyme, and selenium may play roles in enhancing antioxidant defense systems [19]. To explore the effect of Se-PC PDT on the expression of GPx-1 and GPx-4, LLC cells were treated with 150 μg/mL Se-PC PDT and analyzed by western blot analysis after irradiation for 12 h. As shown in Figure 2F, there were almost no changes with Na2SeO3, Na2SeO3+PC or Se-PC treatment. However, an obvious decrease in the expression of GPx-1 and GPx4 was detected after irradiation in all PDT treatment groups.

### Inhibitory effect of Se-PC PDT on tumor-bearing mice

Using subcutaneous tumors in laboratory mice is a very common method among investigators to test antitumor immunity effects in response to PDT treatment because mice have intact immune systems.

The tumor-bearing mice, which were subcutaneously injected with LLC-luc cells, were divided into the following 5 groups: control, laser only, PC PDT (PC-la), Na _2_SeO _3_+PC PDT (NaSe+PC-La) and Se-PC PDT, which were then exposed to laser light of 630 nm at 100 J/cm ^2^ for 4 h after injection with 3 mg PC by the side of the tumor with or without selenium. At the end of the experiment, we performed *in vivo* imaging of tumor-bearing mice using a multifunction imager, at which point the tumor was dissociated, and the volume was recorded.

**Figure 3:**
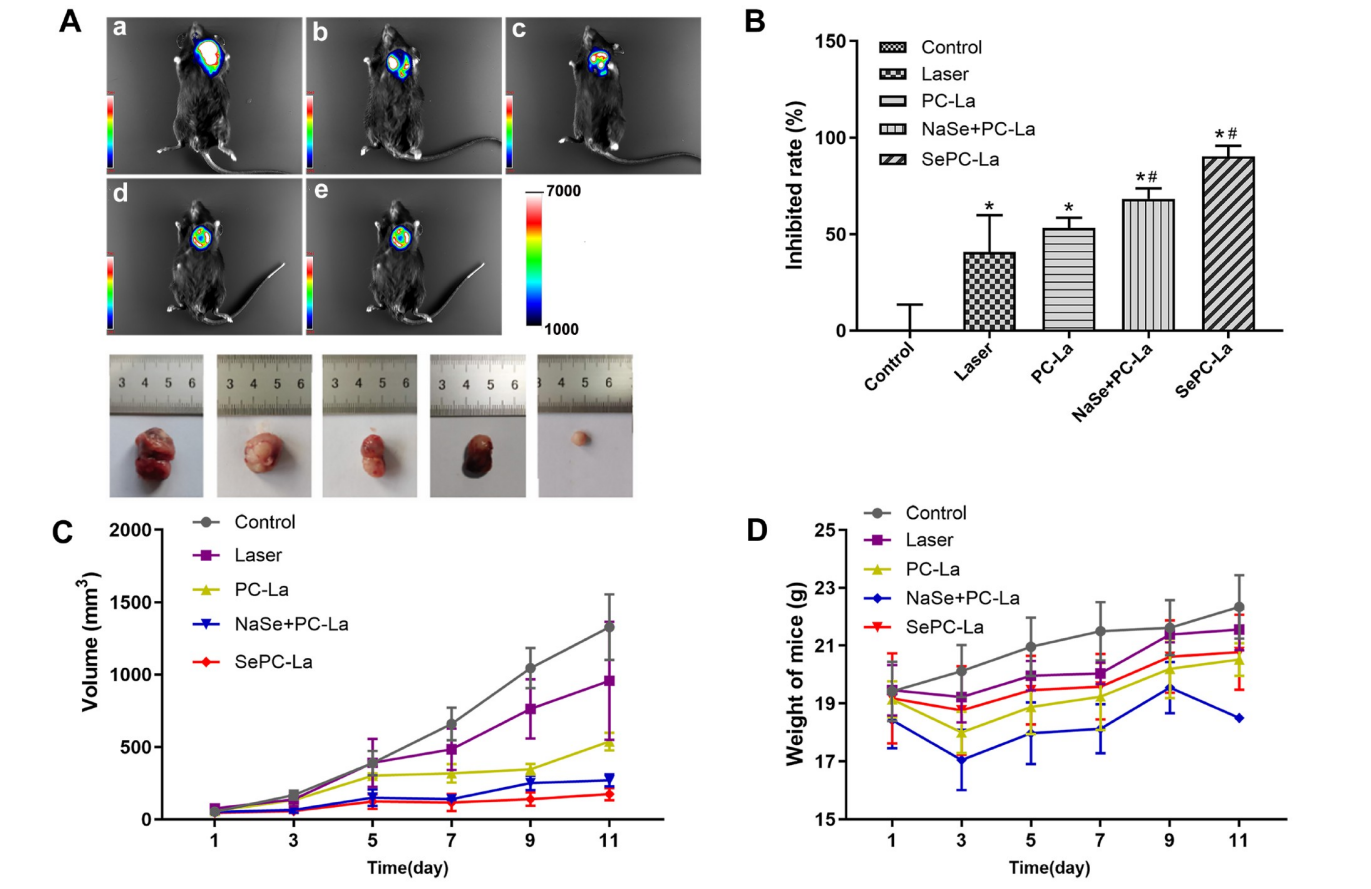
Figure 3 Inhibitory effect of PDT treatments on tumor growth (A) In vivo imaging of tumor-bearing mice. (a) Control; (b) Laser group; (c) PC-La group; (d) NaSe+PC-La group; (e) SePC-La group. (B) Inhibition rate of tumor growth in different treatment groups at the end of the experiment. (C) Tumor volume change in mice over 11 days. (D) Weight change in mice over 11 days.

Based on the luminescence of the *in vivo* tumor imaging and dissociated tumor volume, we demonstrated that the inhibition rate of Se-PC PDT was the most obvious, reaching 90.1% among the PDT treatment groups, followed by the Na _2_SeO _3_+PC PDT group, the PC PDT group and the laser group by 68.3%, 53.1% and 40.7%, respectively. Analysis of body weight and health status showed that phycocyanin mixed with sodium selenite caused weight extenuation, exhibiting obvious side effects on mice; however, there were no changes in response to Se-PC PDT or PC PDT treatment compared to the control group ( Figure 3).

### Metastasis inhibition in response to Se-PC PDT in tumor-bearing mice

**Figure 4:**
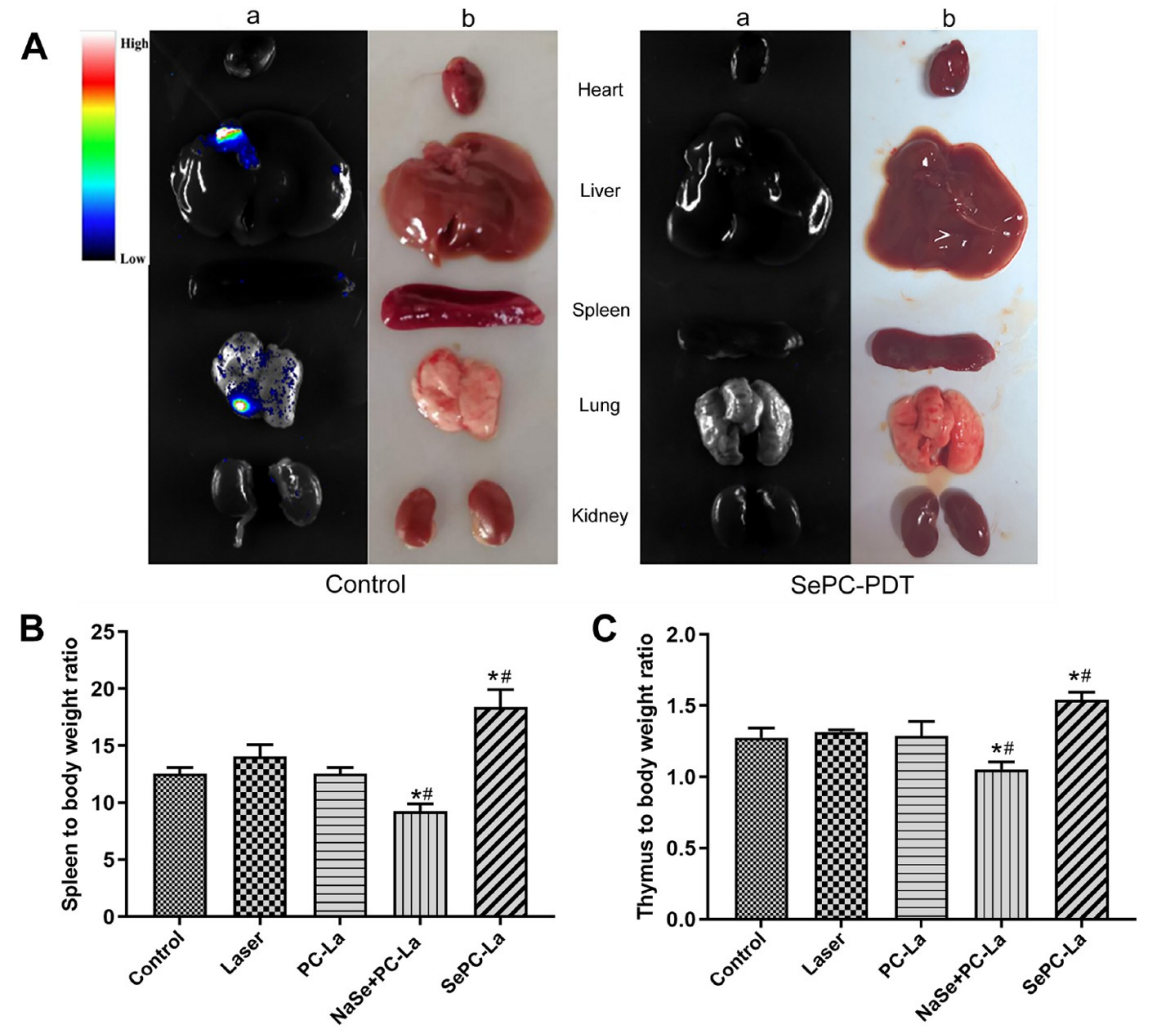
Figure 4 Metastasis inhibition of Se-PC PDT in tumor-bearing mice (A) Metastasis observation in the tissues of LLC-luc tumor-bearing mice. (B) Spleen index. (C) Thymus index. *P<0.05 vs control, #P<0.05 vs PC-PDT (n=9).

To explore the metastasis of tumor cells in mice with or without PDT treatment, we dissociated the organs from LLC-luc tumor-bearing mice and imaged them using a multifunction imager. As shown in Figure 4A, after subcutaneous injection with LLC-luc cells in mice for 18 days, a large area of luminescence was observed in liver and lung tissues, and minimal luminescence was also observed in spleen and kidney. However, no luminescence was detected in normal tissues, including the heart, liver, spleen, lung and kidney, when LLC-luc tumor-bearing mice were treated with the three PDT regimens.

The thymus index and spleen index can be used to evaluate the PDT effect on the immune system of mice. Compared to the control group, Se-PC PDT treatment increased not only the spleen index but also the thymus index, which could be diluted by PC PDT plus Na _2_SeO _3_ treatment, suggesting a reduced effect of Na _2_SeO _3_ on organism immunity ( Figure 4B,C). Our observations revealed that Se-PC PDT treatment inhibited tumor metastasis by enhancing organism immunity but not in response to PC PDT plus Na _2_SeO _3_ treatment.

### Effect of Se-PC PDT on the antioxidase activity and immune response in normal tissues

We assessed the antioxidant enzyme activity of GSH-Px, SOD and MDA in the liver and lung tissues of mice treated with PC PDT, Se-PC PDT and PC PDT plus Na _2_ SeO _3_.

**Figure 5:**
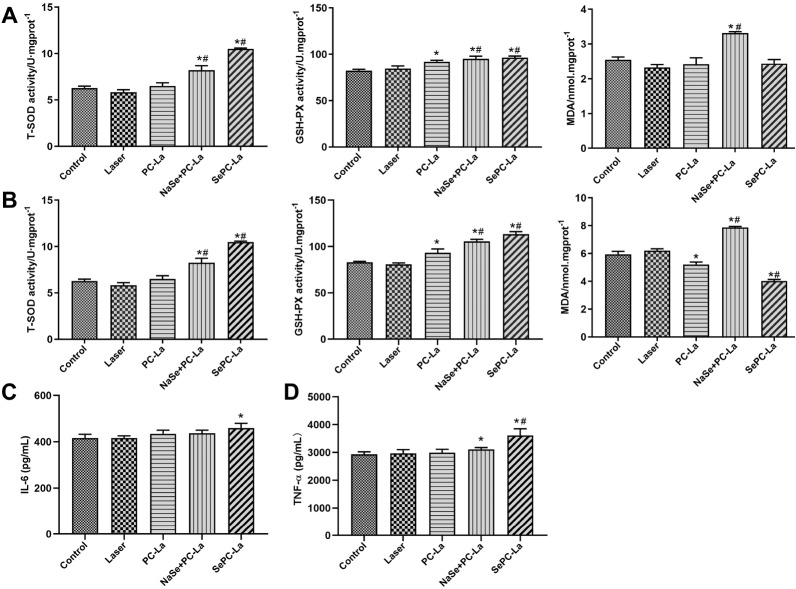
Figure 5 Effect of PDT regimens on antioxidase activity and immune response (A) The antioxidase activity of the liver. (B) The antioxidase activity of the lung. (C) Expression level of IL-6 in serum. (D) Expression level of TNF-α in serum. *P<0.05 vs control, #P<0.05 vs PC-PDT (n =9).

As shown in Figure 5, control and laser treatment alone showed no differences in either liver or lung tissues. The SOD and GSH-Px enzyme activities of Se-PC PDT and PC PDT plus Na _2_SeO _3_ treatment were higher than those of the PC PDT and control groups, indicating that the enzyme activities of GSH-Px and SOD were linked to the addition of selenium.

The results showed that MDA levels in the PC PDT plus Na _2_SeO _3_ group were higher than those of the control group, which were not changed in the PC PDT or Se-PC PDT groups ( Figure 5A) and were even lower than those of the control group ( Figure 5B), suggesting that Na _2_SeO _3_ potentiated membrane damage by the production of lipid peroxide in the liver and lung, and the organic selenium in PC (Se-PC) was safer than inorganic selenium (Na _2_SeO _3_) for normal tissues.

As a core cytokine, IL-6 plays an important role in the acquired immune response by stimulating antibody production and effector T-cell development. TNF-α is mediated through nearly all of the TNF-α receptors on tumor cells and many other cells. High doses of locoregional TNF-α can cause hemorrhagic necrosis via selective destruction of tumor blood vessels by generating specific T-cell antitumor immunity.

In our experiment, Se-PC PDT treatment in mice significantly increased the expression levels of IL-6 and TNF-α in serum to increase the immune response. However, there were almost no changes in response to other PDT treatments except for weakly higher levels of TNF-α in the PC PDT plus Na _2_SeO _3_ group, indicating the important role of selenium in antitumor immunity.

### Cell death type analysis in response to Se-PC PDT tumor treatment

To explore the type of cell death occurring in PDT-treated tumors, we analyzed cellular structure using histological sectioning, transmission electron microscopy (TEM) and expression levels of related genes.

**Figure 6:**
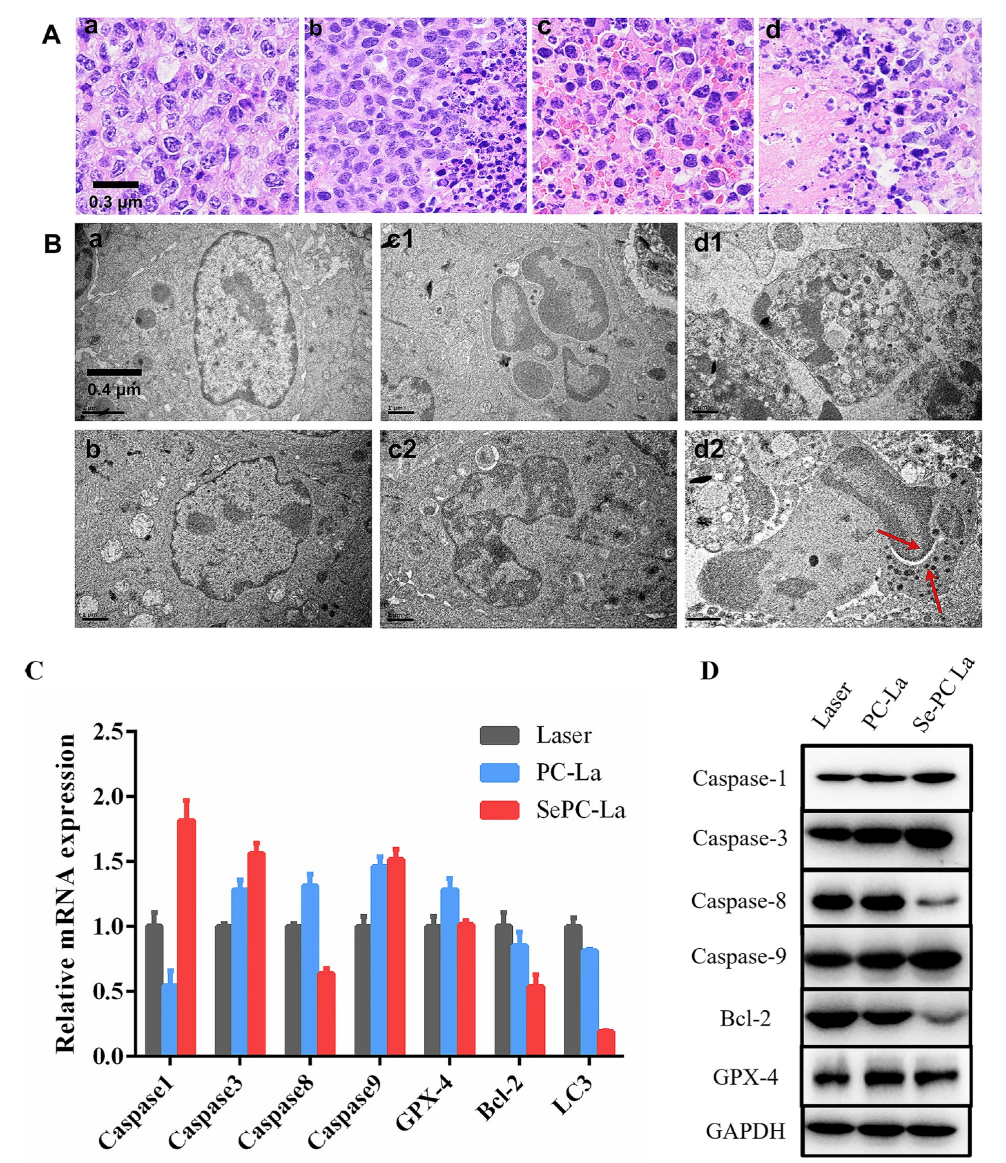
Figure 6 Cell death analysis of Se-PC PDT-treated tumors (A) Paraffin section of the tumor (400×). (a) Control; (b) Laser-only; (c) PC PDT; (d) Se-PC PDT. (B) TEM section of the tumor. (a) Control; (b) Laser-only; (c1 and c2) PC PDT; (d1 and d2) Se-PC PDT. (C) Expression levels of cell death-related genes determined by qRT-PCR. (D) Expression levels of cell death-related proteins determined by western blot analysis.

Tumor pathological sections showed large areas of apoptosis in the PC PDT treatment (PC-La) group and a partial area of apoptosis in the Se-PC PDT group. We also observed cellular necrosis in the laser only group outside of the tumor and in the Se-PC PDT group, which also induced hematocyte breaking compared to the PC PDT group ( Figure 6A).

The TEM images revealed that PC PDT-treated cells exhibited cell membrane and organelle integrity ( Figure 6B) and a small number of phagocytic vacuoles ( Figure 6B). However, there was more digested chromatin, inflated mitochondria and incomplete cell membranes in the Se-PC PDT-treated cells ( Figure 5B) than in the control and laser only groups ( Figure 6B). In particular, the phagocytosis of apoptotic nuclei by a neutrophil is clearly shown in Figure 5B.

Morphological observations revealed that tumor cells treated with Se-PC PDT exhibited cellular necrosis, and those inside the tissue exhibited apoptotic nuclei and vacuoles in the cytoplasm in response to Se-PC PDT treatment. To further identify the cell death type, we also assessed the expression levels of related genes by qRT-PCR and western blot analysis.

Caspase-8 is the initiator caspase of extrinsic apoptosis and inhibits necroptosis. Caspase-1 is a typical marker of cell pyroptosis, and Caspase-9 is an initiator caspase of the mitochondria-dependent apoptosis pathway [20]. Bcl-2 is considered an important antiapoptotic protein [21], and LC3 and GPX-4 are typical markers for autophagy and ferroptosis [22].

As shown in Figure 6C, PC PDT treatment decreased the gene expression levels of Caspase-1, LC3, and Bcl-2 and increased the levels of GPX-4, Caspase-8, Caspase-9 and Caspase-3, indicating the classic cell death type of caspase-dependent apoptosis by inhibition of ferroptosis to avoid lipid peroxide for the cell membrane. However, Se-PC PDT enhanced the gene expression levels of Caspase1, Caspase-9 and Caspase-3 and reduced the expression levels of LC3, Caspase-8, and Bcl-2, exhibiting typical characteristics of pyroptosis and inhibition of autophagy.

To further confirm the role of selenium in PDT treatment, we also detected the levels of Caspase-1, Caspase-3, Caspase-8, Caspase-9, Bcl-2 and LC3 by western blot analysis. As shown in Figure 6D, both PC PDT and Se-PC PDT treatment increased the activity of cleaved Caspase-1, Caspase-3 and Caspase-9 and significantly reduced the activity of Bcl-2 and cleaved LC3, in which Se-PC PDT exhibited higher increased or reduced levels than PC PDT treatment. We also found that Se-PC PDT but not PC PDT treatment increased the level of Caspase-8 and decreased the level of VEGF.

Therefore, our results demonstrated that tumors treated with Se-PC PDT exhibited cellular pyroptosis and that those treated with PC PDT exhibited apoptotic nuclei. Selenium plays an important role in increasing the expression levels of Caspase-1, Caspase-3, Caspase-8, and Caspase-9 and decreasing the expression levels of Bcl-2 and LC3, which promote the development of cellular pyroptosis and apoptosis and increase the rate of tumor cell death.

### Transcriptome analysis of Se-PC-PDT on the tumors of mice

**Figure 7:**
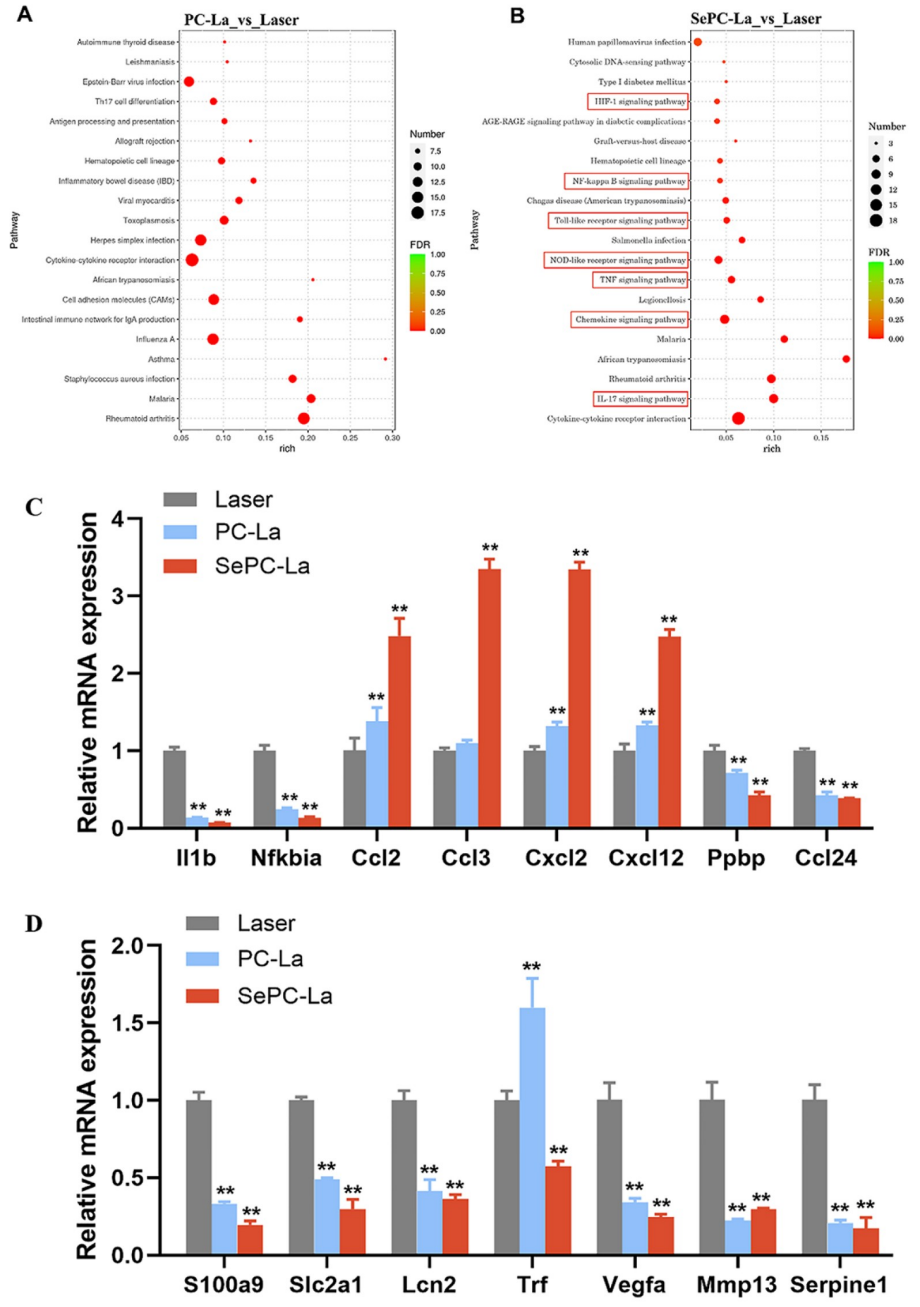
Figure 7 Main pathways according to KEGG analysis and expression levels of associated genes (A) PC-la vs laser. (B) SePC-La vs laser. (C) Gene expression levels of inflammatory factors. (D) Expression levels of genes related to cell metabolism, angiogenesis and metastasis.

To further explore the molecular mechanism of the therapeutic effect of selenium enrichment, we performed high-throughput sequencing on mRNAs of the tumor samples from three groups, including the laser only (Laser), PC PDT (PC-La), and Se-PC PDT (SePC-La) groups, in triplicate. According to the comparison of the top 20 enriched KEGG pathways between PC-La vs laser and SePC-La vs laser ( Figure 7A,B), the differential pathways in the Se-PC PDT group were mainly involved in seven signaling pathways ( Table 3). They were the IL-17 signaling pathway, chemokine signaling pathway, TNF signaling pathway, NOD-like receptor signaling pathway, Toll-like receptor signaling pathway, NF-κB signaling pathway and HIF-1 signaling pathway.

**Table:** **Table 3** Enriched signaling pathways and the selected genes

Term	*P* value	Gene
IL-17 signaling pathway	1.75×10 ^–08^	*Nfkbia*, *Mmp13*, *Ccl2*, *Il1b*, *Lcn2*, *S100a9* and *Cxcl2*
Chemokine signaling pathway	8.74×10 ^–06^	*Ccl2*, *Ccl3*, *Cxcl2*, *Cxcl12*, *Nfkbia*, *Ccl24* and *Ppbp*
TNF signaling pathway	0.000133998	*Ccl2*, *Il1b*, *Cxcl2* and *Nfkbia*
NOD-like receptor signaling pathway	0.000222121	*Nfkbia*, *Cxcl2*, *Il1b* and *Ccl2*
Toll-like receptor signaling pathway	0.00080114	*Ccl3*, *Nfkbia* and *Il1b*
NF-κB signaling pathway	0.004326816	*Nfkbia*, *Il1b* and *Cxcl12*
HIF-1 signaling pathway	0.005831104	*Trf*, *Serpine1*, *Slc2a1* and *Vegfa*

Based on the analysis of RNA-seq data, the target genes of seven enriched pathways were confirmed, and they were divided into three groups: inflammatory factors ( Figure 7C), cell metabolism, angiogenesis and metastasis ( Figure 7D) after comparison of the gene expression levels among the laser only, PC PDT and Se-PC PDT groups.

Increasing evidence indicates that the tumor microenvironment and tumor-associated inflammation may play critical roles in disease progression. IL-1B is an inflammatory chemokine expressed via the NF-κB (gene Nfkbia) signaling pathway that plays important roles in tumor initiation and metastasis [23].

Chemokines such as CCL2 and G-CSF [ 24– 26], which are secreted by tumor or endothelial cells, are responsible for leukocyte attraction. As a potent chemokine for lymphocytes and monocytes, CCL3 plays a critical role in regulating lymph node homing of dendritic cell subsets and induces antigen-specific T-cell responses [27]. CCL24 (eotaxin-2) is one type of eotaxin [28] and is the primary chemotactic factor for eosinophils. Elevated expression of eotaxins also occurs in tumors and is associated with the recruitment of eosinophils into the tumor niche. Some results have shown that the expression of CXCL2 is associated with NF-κB activation involved in tumor invasion and metastasis [29], and the chemokine CXCL12 recruits immune cells infiltrating cancerous foci [30] and triggers tumor-associated inflammation. Our results indicated that both Se-PC PDT and PC PDT treatment dramatically downregulated the gene expressions of IL-1b, Nfkbia and Ccl24 and upregulated the gene expressions of Ccl2, Ccl3, Cxc12 and Cxcl12, in which Se-PC PDT treatment induced markedly higher expression levels than PC PDT treatment ( Figure 7C). Platelets activate the granulocyte-expressed receptor CXCR2 by secreting the chemokine CXCL7 (Ppbp gene) and recruiting granulocytes, contributing to tumor cell extravasation from the blood [31].

Tumor cells secrete vascular endothelial growth factor VEGF (gene Vegfa), which creates a proangiogenic environment [32]. MMP-13 expression is regulated by Serpine1 (PAI-1), which is known to inhibit the plasminogen activator enzyme and is related to malignancies by influencing tumor invasion, angiogenesis and metastasis [33]. Transcriptional activation of genes that are upregulated by TGF-β is dependent on PAI-1 expression level in an inflammatory environment. Our data showed that both Se-PC PDT and PC PDT treatment markedly downregulated the gene expressions of Vegfa, Ppbp, Mmp13 and Serpine1 to inhibit tumor proliferation and metastasis.

S100a9 induces translocation of the adaptor protein MyD88 from the cytoplasmic space to the receptor in the cell membrane to activate some transcription factors, such as p38 and NF-κB [34]. Solute carriers (SLCs) of the glucose transporter GLUT1 (gene family name Slc2a1) accelerate metabolism as a preeminent actor in tumors [35]. LCN2 (gene family name Lcn2), which binds to iron-loaded catecholate through the LCN2 receptor (LCN2R), increases intracellular iron to protect tumor cells from ROS-induced damage and apoptosis [36]. Transferrin (gene Trf) may be attributed to iron requirements, with iron being an important cofactor of the ribonucleotide reductase enzyme involved in the synthesis of DNA in rapidly dividing cells [37].

In our experiment, both PDT treatment of Se-PC and PC downregulated the gene expressions of S100a9, Slc2a1, and Lcn2 to slow tumor proliferation by regulating cell metabolism and iron binding. However, Se-PC PDT treatment downregulated but PC PDT upregulated the gene expression level of Trf, suggesting that Se-PC PDT rapidly inhibits the proliferation of tumor cells, in contrast to PC PDT treatment.

## Discussion

The antitumor effects of PDT are resulted from the combination of three different *in vivo* mechanisms, namely, direct PDT cytotoxicity to tumor cells, destruction of the tumor microvasculature and induction of an acute local inflammatory response, leading to activation of the host immune system [4]. However, the molecular mechanism of Se-PC PDT, particularly the synergistic effect of PC PDT and selenium, has not been well defined. In this study, we designed three kinds of photosensitizers to determine differences in response to selenium addition: PC, selenium-enriched PC and PC mixed with Na _2_SeO _3._

Our *in vitro* experiment showed that Se-PC PDT could more efficiently reduce the survival rate of the LLC cell line than PC PDT treatment by increasing the level of ROS and decreasing the levels of GPx1 and GPx4. The Chou-Talalay assay confirmed that selenium could enhance the effect of PC PDT with a combination index (CI) value of 0.70, indicating that the combination of selenium and PC PDT exhibits a good synergistic effect against lung cancer cells.

In an *in vivo* experiment, we found that the tumor inhibition rates of the Se-PC PDT, PC PDT plus Na _2_SeO _3_ and PC PDT groups reached 90.4%, 68.3% and 53.1%, respectively. We also demonstrated that Se-PC PDT treatment inhibited tumor metastasis by enhancing organ immunity but not PC PDT plus Na _2_SeO _3_ treatment, which also increased MDA levels in both liver and lung tissues, suggesting that the organic selenium in PC (Se-PC) was more effective against tumors and safer than inorganic selenium (Na _2_SeO _3_) for normal tissues. TEM analysis revealed that tumor treated with Se-PC PDT exhibited cellular pyroptosis and apoptotic nuclei after PC PDT treatment.

Caspase-8 is the molecular switch for apoptosis [38], necroptosis and pyroptosis. Caspase-8 most likely acts upon Caspase-3. When necroptosis is inhibited or the apoptotic pathway is incomplete, pyroptosis is induced. Based on the transcriptome analysis, we summarized the possible molecular mechanism of the Se-PC PDT regimen in tumor-bearing mice. After absorption of PC in tumors and irradiation with laser, ROS are produced in tumor cells to induce extracellular apoptosis by increasing the activity of Caspase-8 and Caspase-3 and decreasing the activity of Bcl-2, an important factor for cell proliferation. Using Se-PC as a photosensitizer in tumors, pyroptosis was the primary type of tumor death by increasing the activity of Caspase-1 and Caspase-3 and reducing the activity of Caspase-8 and Bcl-2. Meanwhile, both Se-PC PDT and PC PDT treatment inhibited tumor initiation and metastasis by downregulating the expression levels of Vegfa, Mmp13 and Serpinel via the HIF-1 pathway and IL-1b and Nfkbia via the NF-κB and TGF-β signaling pathways. Moreover, Se-PC PDT treatment may dilute tumor metabolism and proliferation by depressing the activity of the glucose transporter GLUT1 (gene Slc2a1) and the iron transport-related factors LCN2 (gene Lcn2) and transferrin (gene Trf), which reduce the synthesis of hemoglobin and inhibit ferroptosis.

C-PC-mediated PDT was reported to activate the immune system, including amplification of leukocyte activity [15]. In our experiment, the obvious differentiation between Se-PC PDT and PC PDT treatment was tumor-associated inflammation. ROS also initiated the inflammatory reaction by the NF-κB pathway to secrete chemokines both in tumor cells and the tumor microenvironment, such as CCL2, CCL3, CXCL2, and CXCl12, which were upregulated by Se-PC PDT, and CCL24 and CXC17, which were downregulated by Se-PC PDT in the tumor niche. These chemokines enhanced the recruitment of leukocytes and lymphocytes, which induced an antigen-specific T-cell response to inhibit cell proliferation and metastasis and phagocytose dead cells in the tumors.

**Figure 8:**
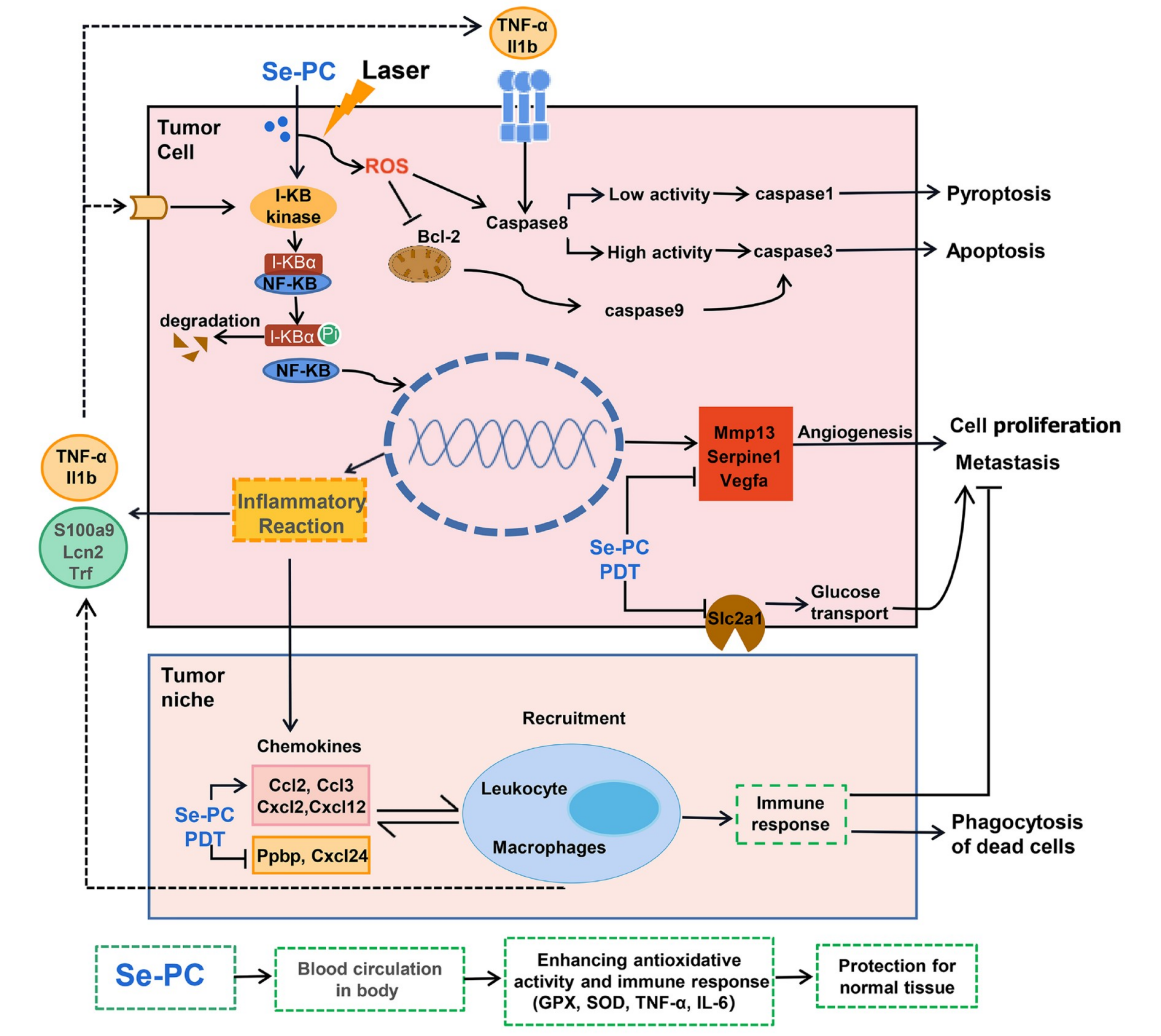
Figure 8 Molecular mechanism analysis of Se-PC-PDT-treated tumors

The biological effect of selenium is dose dependent. In the range of certain concentrations, selenium can promote the production of GSH-Px, but [GSH+SeO _3_^2^] will be produced and cytotoxic to the cell at higher doses when O _2_ is present. In our experiment, ROS were produced after irradiation and triggered cell death, including apoptosis and pyroptosis, when tumors were treated with higher concentrations of Se-PC. Simultaneously, the Se-PC peptide can enter the blood circulation, and irradiation promotes the synthesis of antioxidant enzymes and the immune response by the IL-6/TNF-α pathway to protect normal tissues against tumor metastasis ( Figure 8).

In summary, the antitumor effect of Se-PC PDT may address ROS-induced tumor-associated inflammation, and Caspase-8 switches cell death by inhibiting cell proliferation and angiogenesis, diluting cell metabolism and enhancing antioxidant activity and immune capacity in normal tissues. Taken together, our study provides a successful strategy to enhance the phototherapy efficacy by combining PDT and Se-PC from Spirulina platensis against lung cancer.
